# Atypical presentation of mature cystic teratoma (“floating balls”)

**DOI:** 10.1590/0100-3984.2015.0155

**Published:** 2017

**Authors:** Ana Paula Barroso Pazinatto Espindola, Viviane Brandão Amorim, Hilton Augusto Koch, Paulo Roberto Valle Bahia, Márcio V. P. Almeida

**Affiliations:** 1 Pontifícia Universidade Católica do Rio de Janeiro (PUC-Rio), Rio de Janeiro, RJ, Brazil.; 2 Centro Estadual de Diagnóstico por Imagem do Rio de Janeiro (CEDI), Rio de Janeiro, RJ, Brazil.

**Figure 1:**
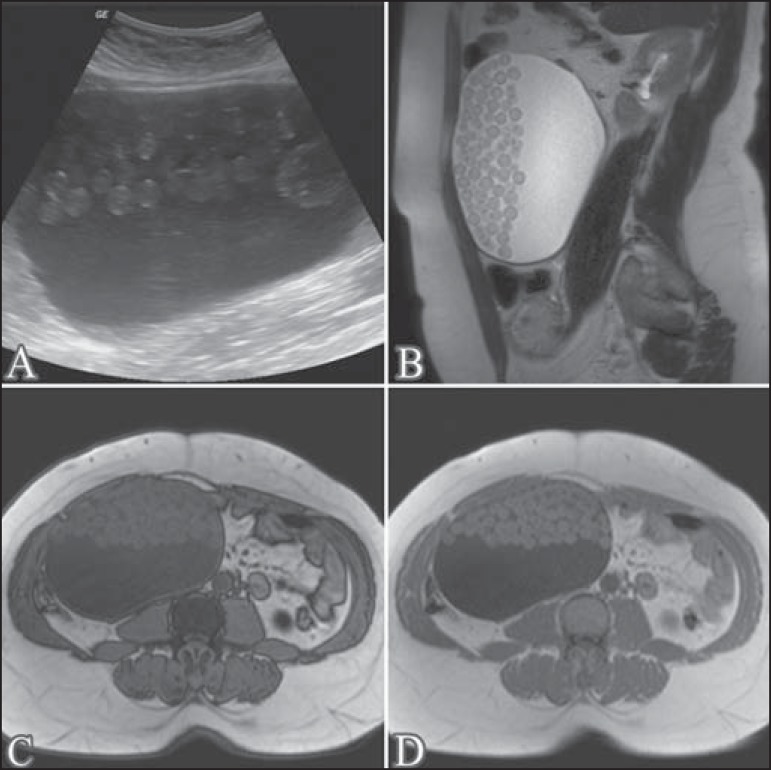
Ultrasound (**A**); sagittal T2-weighted MRI sequence (**B**); out-of-phase T1-weighted gradient-echo MRI sequence (**C**); and in-phase T1-weighted gradient-echo MRI sequence (**D**). Note the expansile cystic lesion with heterogeneous content, containing numerous oval formations that were hyperechoic on the ultrasound and showed intermediate signal intensity in the T1- and T2-weighted sequences, with no evidence of signal loss in the out-of-phase T1-weighted gradient- echo sequence.

Dear Editor,

A 43-year-old female patient with no known diseases sought medical attention complaining of increased abdominal volume. The patient underwent ultrasound and subsequent magnetic resonance imaging (MRI) of the pelvis (Figure 1), which showed an expansile cystic lesion, with heterogeneous content, measuring 16.0 × 16.0 × 10.0 cm and containing numerous oval formations of various sizes. The lesion was hyperechoic on ultrasound and mobile upon a change in patient position. The oval formations showed intermediate signal intensity on T1- and T2-weighted MRI scans, with no evidence of signal loss in fat-saturated sequences or signal drop on an out-of-phase T1-weighted gradient-echo sequence. These imaging findings, although uncommon, are pathognomonic of mature cystic teratoma (MCT). The patient underwent surgery, and the diagnosis was confirmed by histopathological analysis of the surgical specimen.

Also known as a dermoid cyst, MCT is the most common benign ovarian tumor, accounting for 10-25% of cases in adult patients and 50% of those in pediatric patients^(1-3)^. MCTs are typically asymptomatic and slow-growing^(1,3)^. They are usually seen in women of reproductive age and are rarely diagnosed before puberty. Its growth ceases at menopause^(4-7)^. An MCT typically contains well-differentiated tissues of the three germ layers^(1,5)^: the ectoderm, (derived from the skin and neural tissues); the mesoderm (osteomuscular and adipose tissues); and the endoderm (ciliated and mucinous epithelium). The diversity of tissues in teratomas results in a wide variety of characteristics in imaging studies.

In most cases, pelvic tumors do not present imaging features that are considered diagnostic^(8-12)^. However, MCTs often present typical imaging features, which facilitate the diagnosis. Among such features, one of the most common is that of a fatty tumor^(3)^. In such cases, the most common ultrasound finding is that of a cystic mass with an echogenic tubercle (a Rokitansky nodule), presenting posterior acoustic shadowing secondary to calcifications, strands of hair, or foci of fat^(3,5,7)^.

Characteristic findings on computed tomography include areas of fat attenuation, with or without foci of calcification. On MRI, the fat seen within the lesion produces a hyperintense signal on T1-weighted images and signal loss in fat-saturated sequences^(3,5,7)^. In rare cases, the presentation of MCT is atypical, which can be a diagnostic challenge for radiologists^(2,6)^. Multiple small floating spheres within a large cyst, as observed in the case presented here, is one of those rare presentations, known as the “floating ball” presentation^(4,6)^. Histologically, the spheres are composed of keratin, fibrin, hemosiderin, sebaceous debris, hair, and fat, in variable proportions^(2,6,13)^. Although the mechanism of formation of these spheres has yet to be clarified, it is speculated that it involves aggregation of sebaceous material around a nidus^(2,4,14)^. The mobility of the spheres is due to their low density relative to the other content of the cyst^(2,4,6)^. A finding of multiple floating spheres within a single large cyst has not been reported for other types of tumors and is therefore considered pathognomonic of MCT^(2,4,6,14-16)^.
